# Supplementary material for: Altered chemistry of oxygen and iron under deep Earth conditions
Source: Nat Commun. 2019 Jan 11;10:153. doi: 10.1038/s41467-018-08071-3 (PMC6329810; doi:10.1038/s41467-018-08071-3)
Supplement: Supplementary file 1 — Supplementary Information [file 41467_2018_8071_MOESM1_ESM.pdf]

# Altered chemistry of oxygen and iron under deep Earth conditions

Liu et al.

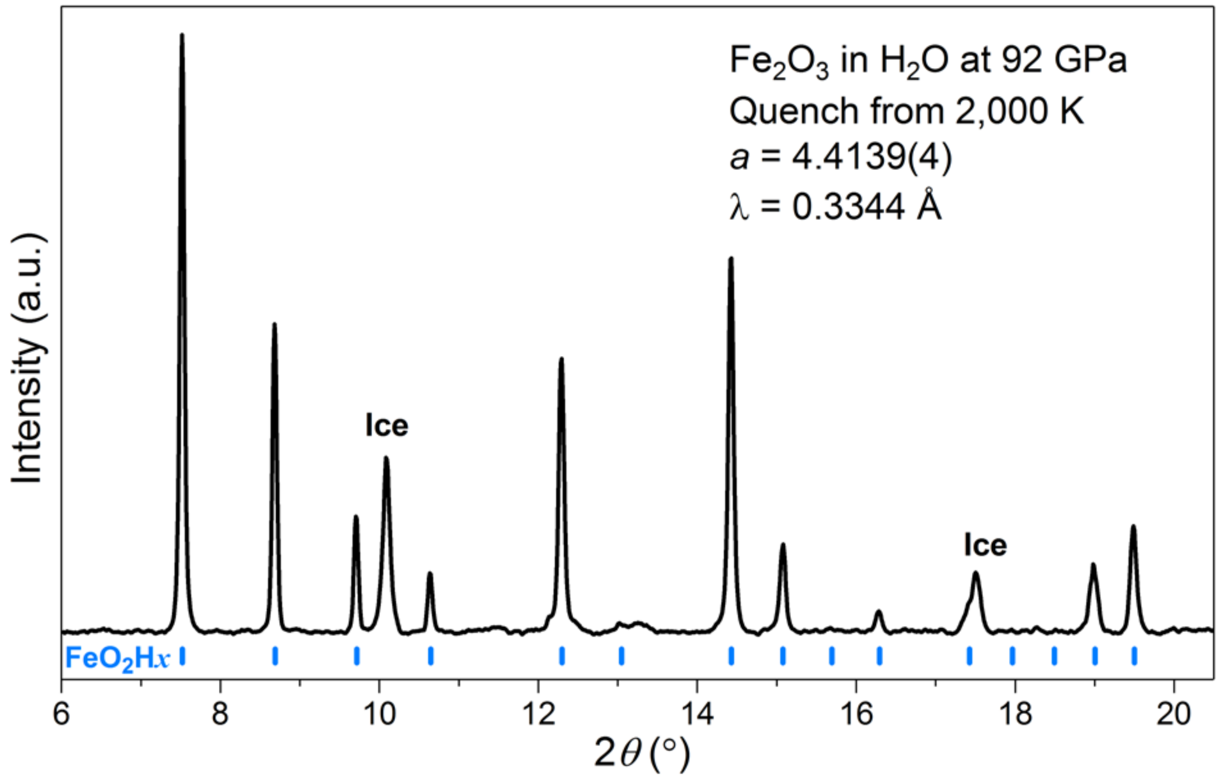


**Supplementary Figure 1. Representative X-ray diffraction pattern of reaction products of $Fe_2O_3$ with excess $H_2O$ at 92 GPa and quench from 2,000 K.** In this run, the whole Sample # N1 has been scanned by XRD after laser heating for 3 hours at 92 GPa. It is found that there was not any detectable amount of starting materials $Fe_2O_3$ left in the sample chamber.

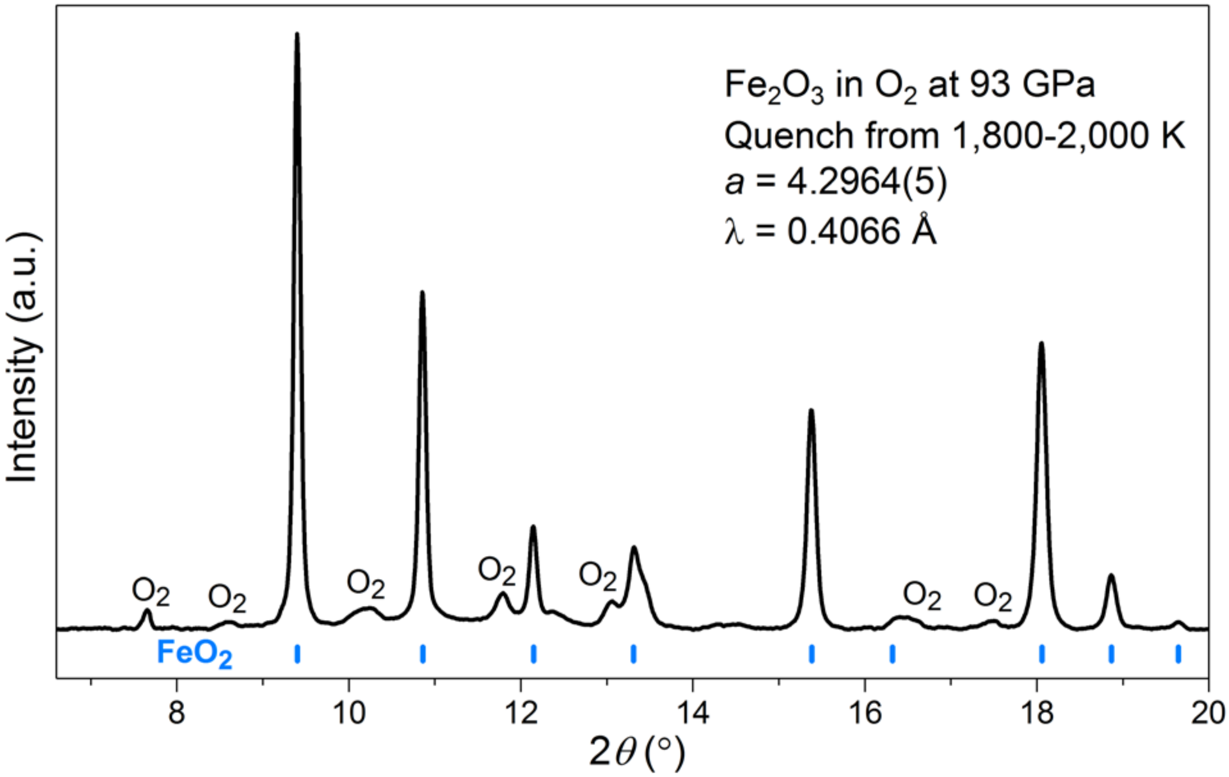


**Supplementary Figure 2. Representative X-ray diffraction pattern of reaction products of $Fe_2O_3$ with excess oxygen at 93 GPa and quench from 1,800-2,000 K.** In this run, the whole Sample # N2 has been scanned by XRD after laser heating for 3 hours at 93 GPa. It is found that there was not any detectable amount of starting materials $Fe_2O_3$ left in the sample chamber.

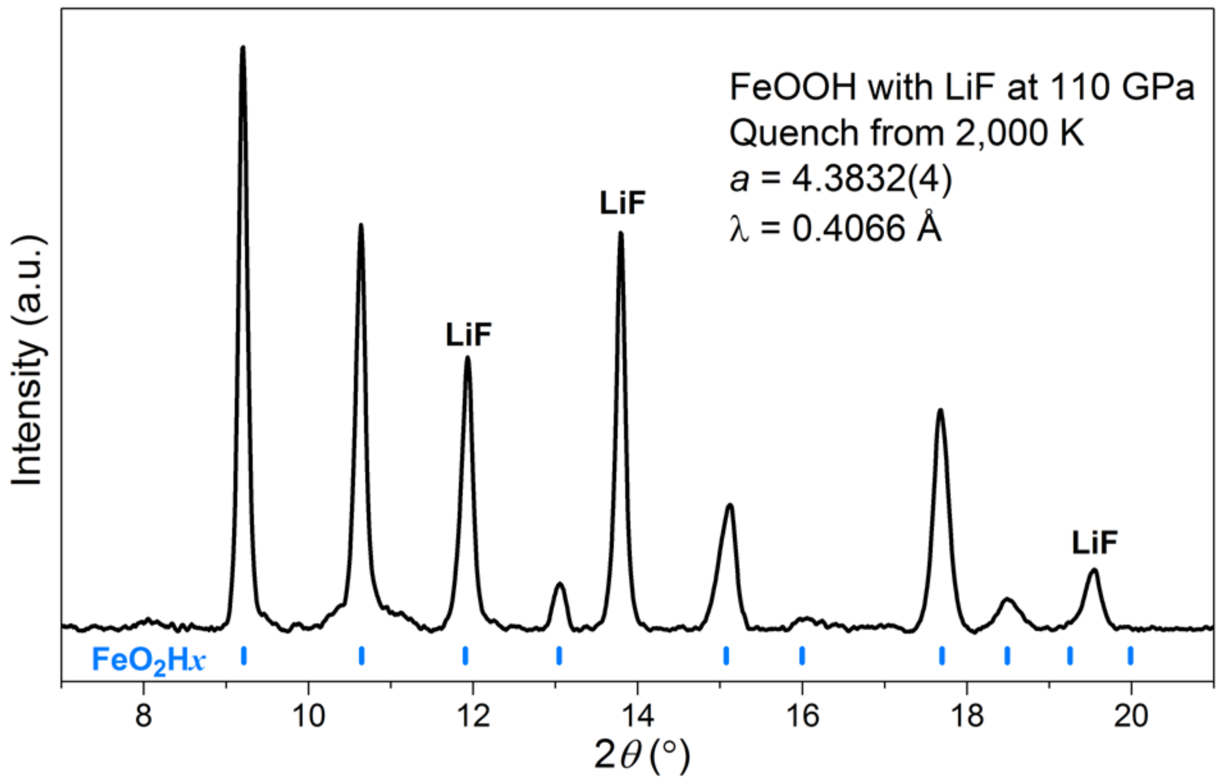


**Supplementary Figure 3. Representative X-ray diffraction pattern of reaction products of goethite FeOOH at 110 GPa and quench from 2,000 K.** LiF was deliberately used as the pressure-transmitting medium as it does not contain oxygen. In this run, the whole Sample EEL#1 has been scanned by XRD after laser heating for 3 hours at 110 GPa. It is found that there was not any detectable amount of starting materials FeOOH left in the sample chamber.

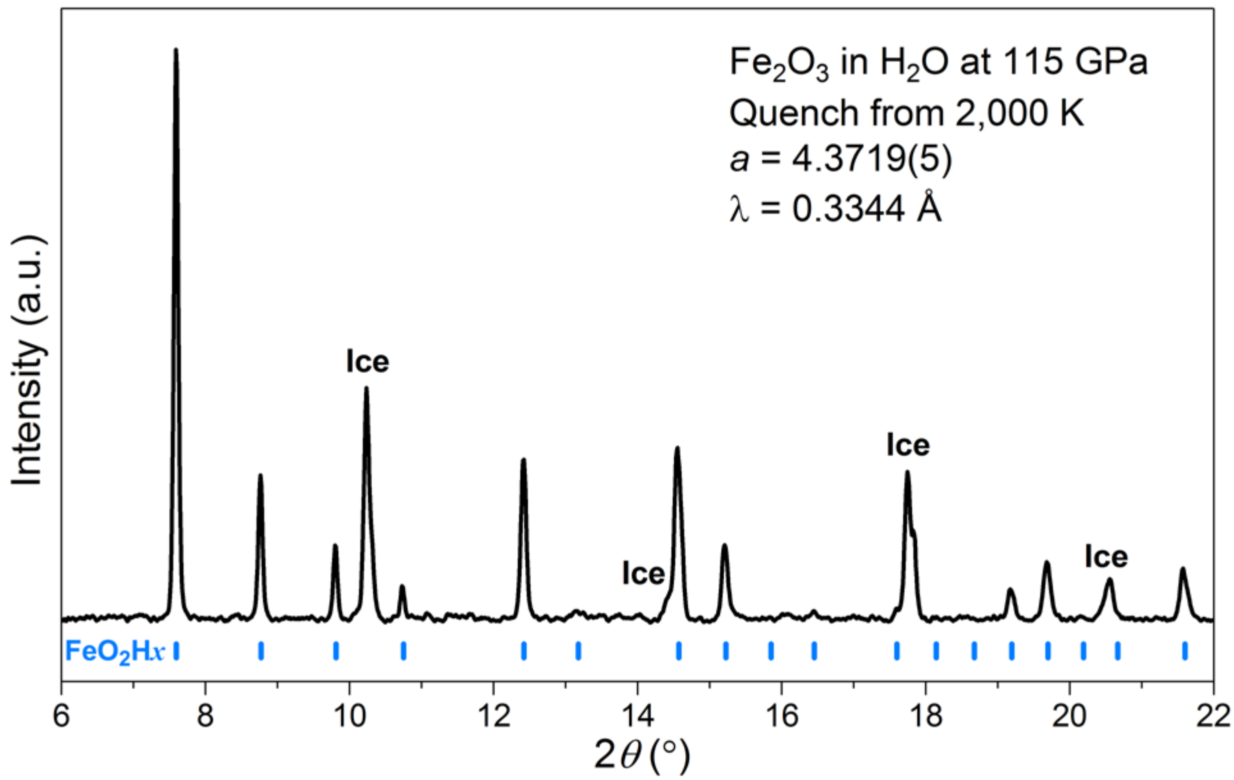


**Supplementary Figure 4. Representative X-ray diffraction pattern of reaction products of $Fe_2O_3$ with excess $H_2O$ at 115 GPa and quench from 2,000 K.** In this run, the whole Sample CC#96 has been scanned by XRD after laser heating for 2 hours at 115 GPa. It is found that there was not any detectable amount of starting materials $Fe_2O_3$ left in the sample chamber.

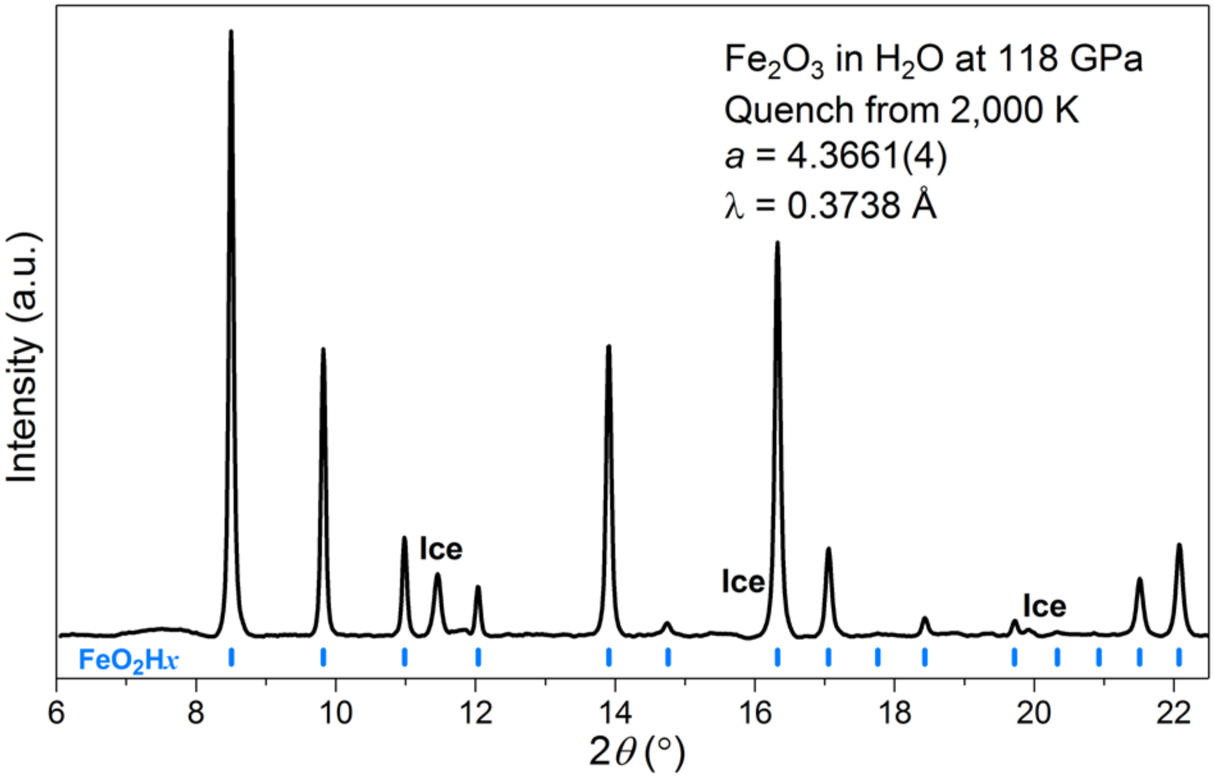


**Supplementary Figure 5. Representative X-ray diffraction pattern of reaction products of $Fe_2O_3$ with excess $H_2O$ at 118 GPa and quench from 2,000-2,200 K.** In this run, the whole Sample CC#233 has been scanned by XRD after laser heating for 2 hours at 118 GPa. It is found that there was not any detectable amount of starting materials $Fe_2O_3$ left in the sample chamber.

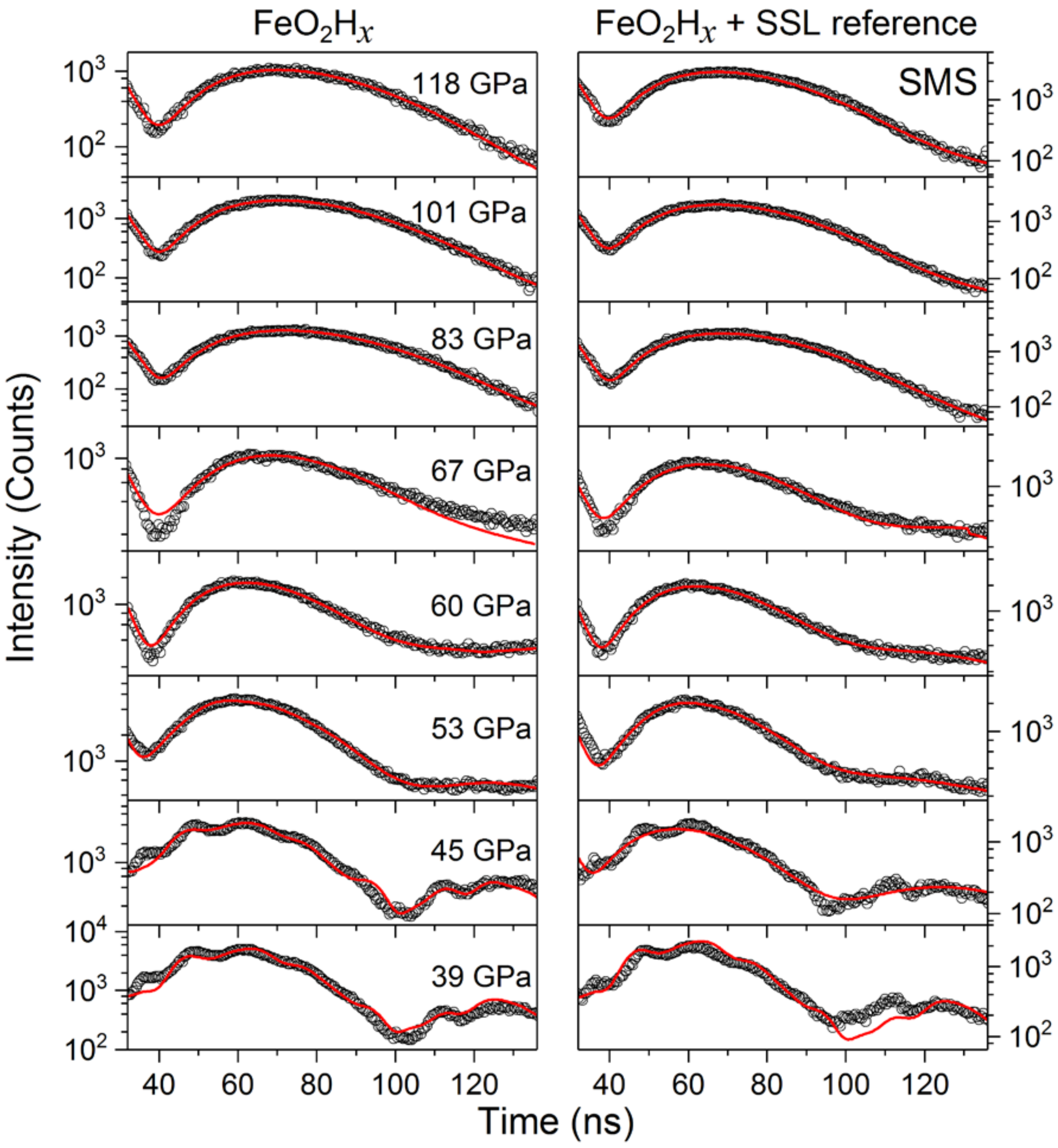


**Supplementary Figure 6. Synchrotron Mössbauer spectra of pyrite-structured $FeO_2H$*x* as a function of pressure at 300 K.** Circles: experimental SMS spectra; red solid lines: modeled spectra using CONUSS program. Stainless steel (SSL) foil was used as a reference to derive isomer shifts of iron in $FeO_2H$*x* in the first run of SMS experiments

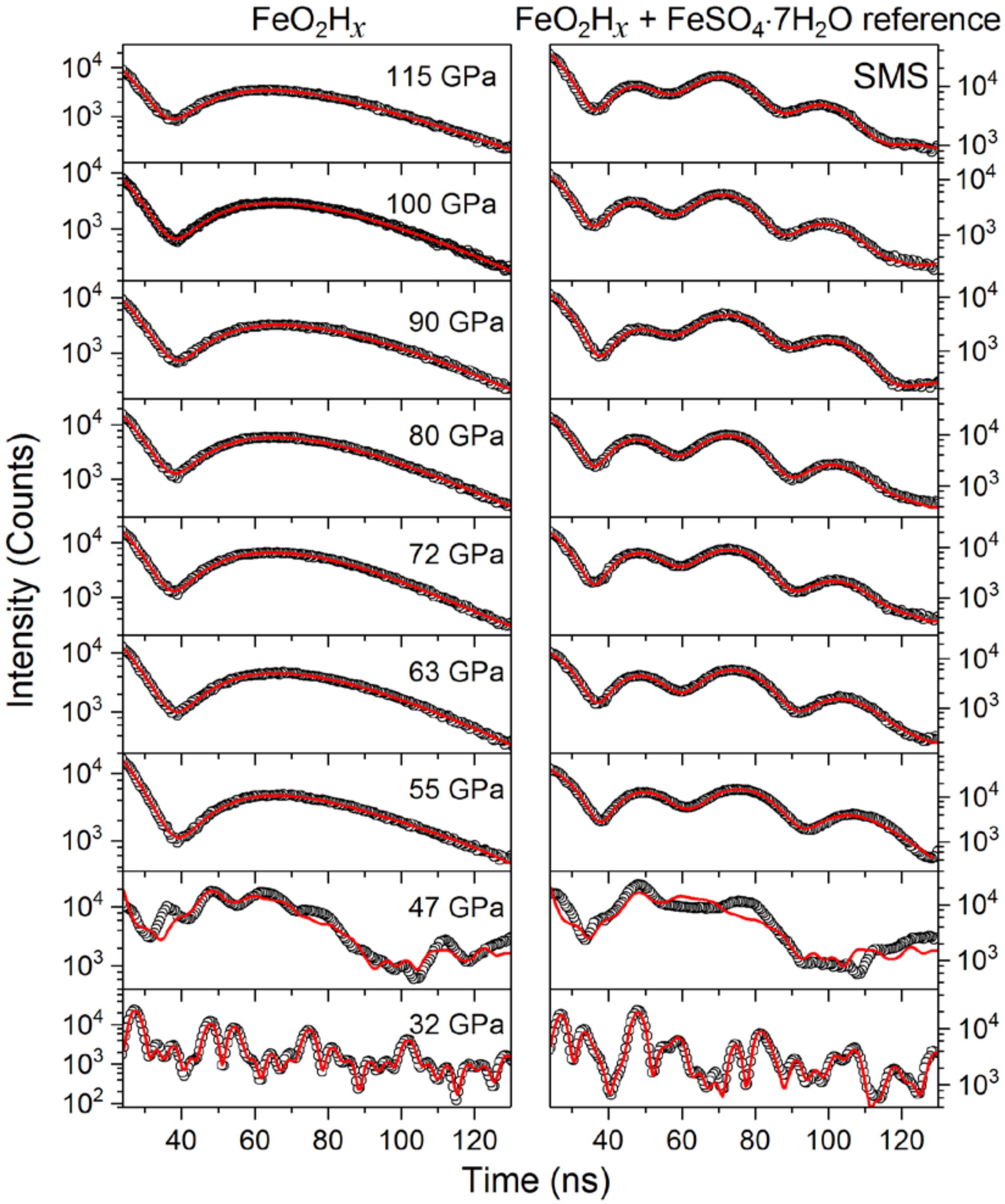


**Supplementary Figure 7. Synchrotron Mössbauer spectra of pyrite-structured $FeO_2H_x$ as a function of pressure at 300 K.** Circles: experimentally measured SMS spectra; red solid lines: modeled spectra using CONUSS program. Ferrous sulfate heptahydrate ($FeSO_4 \cdot 7H_2O$) was used as a reference to derive isomer shifts of iron in $FeO_2H_x$ in the second run of SMS experiments**.**

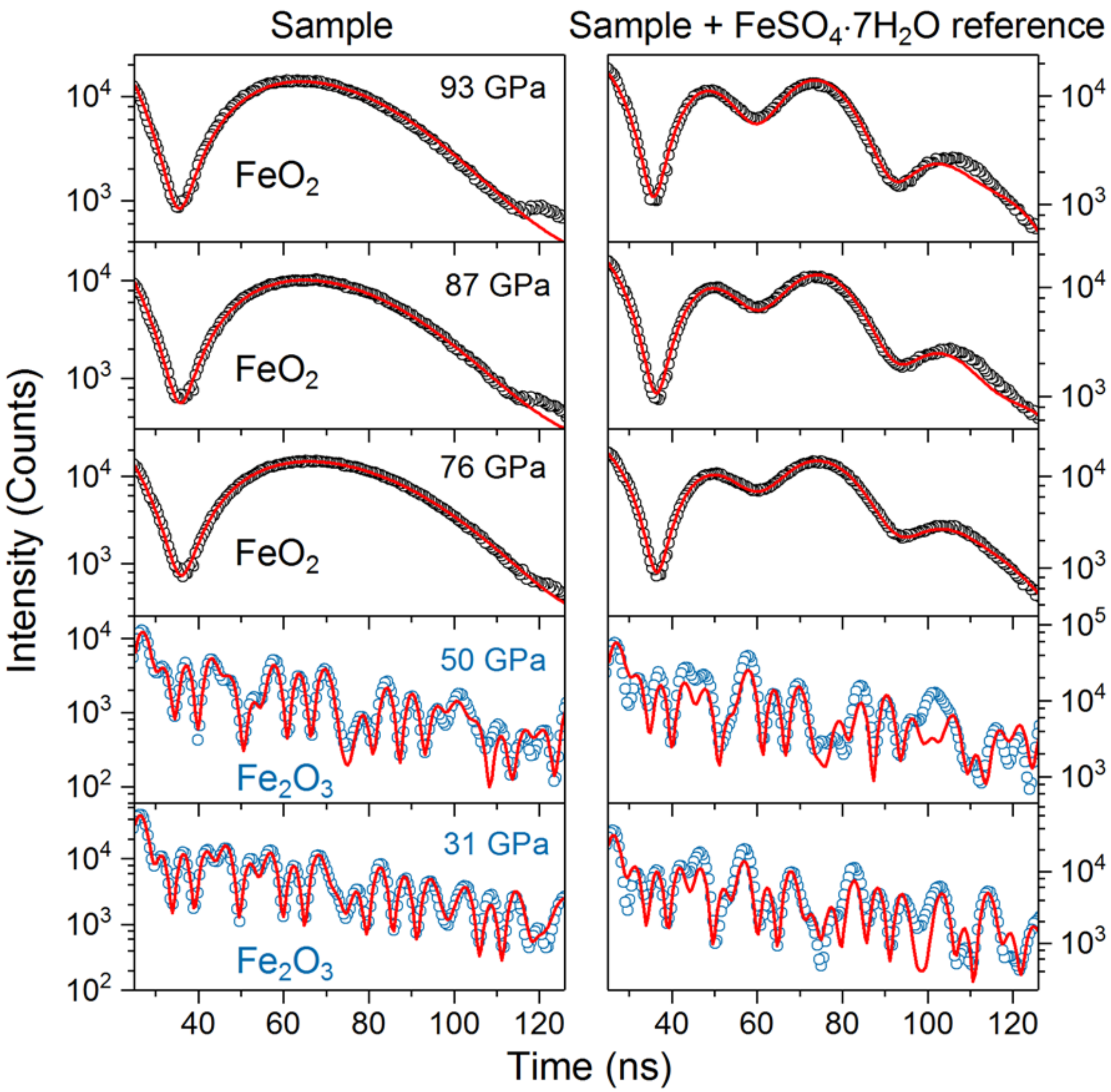


**Supplementary Figure 8. Synchrotron Mössbauer spectra of pyrite-structured $FeO_2$ and $Fe_2O_3$ at high pressures and 300 K.** Circles: experimentally measured SMS spectra; red solid lines: modeled spectra using CONUSS program. Ferrous sulfate heptahydrate ($FeSO_4{\cdot}7H_2O$) was used as a reference to derive isomer shifts of iron in pyrite-structured $FeO_2$ and $Fe_2O_3$.

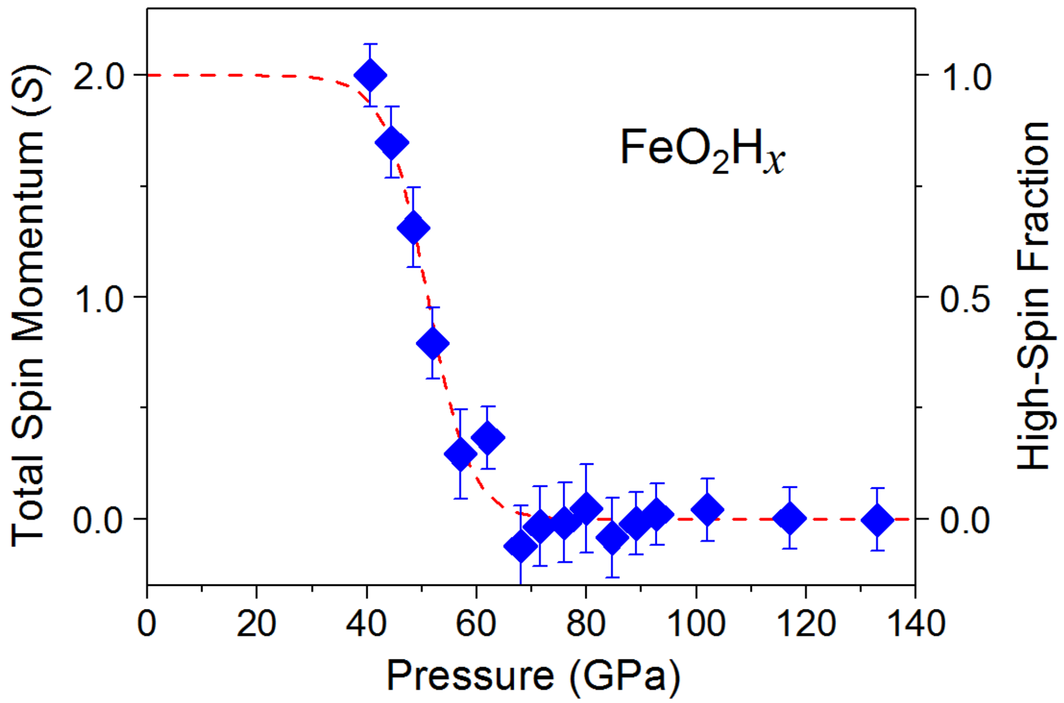


**Supplementary Figure 9. Total spin momentum and high-spin fraction of pyrite-structured $FeO_2H_x$ as a function of pressure derived from the XES measurements at 40–133 GPa and 300 K.** Blue diamonds: experimental measurements; red line: fitting results. Vertical ticks represent the error for the high-spin fraction. Errors are calculated using standard error propagations from our experimental data.

**Supplementary Table 1. Experimental conditions for synthetic samples of $FeO_2H_x$.**

| Sample # | Synthetic Conditions | Hydrogen ($x$) | Experiments |
|---|---|---|---|
| N1 | $Fe_2O_3$ + $H_2O$ at 92GPa and 2,000K for 3 h | 0.5-0.7 | XAS, XES |
| N2 | $Fe_2O_3$ + $O_2$ at 93GPa and 1,800-2,000K for 3 h | 0 | XAS, SMS |
| EEL#1 | FeOOH in LiF at 110GPa and 2,000K for 3 h | 0.6-0.8 | XRS |
| CC#96 | $Fe_2O_3$ + $H_2O$ at 115GPa and 2,000-2,200K for 2 h | 0.5-0.7 | SMS |
| CC#233 | $Fe_2O_3$ + $H_2O$ at 118GPa and 2,000-2,200K for 2 h | 0.5-0.7 | SMS |
